# Navigate Your Health: A Case Study of Organisational Learnings from an Integrated Care Pilot for Children and Young People in Care

**DOI:** 10.5334/ijic.5659

**Published:** 2021-08-09

**Authors:** Perrin Moss, Rebecca O’Callaghan, Andrea Fisher, Craig Kennedy, Frank Tracey

**Affiliations:** 1 Bachelor of Business, Bachelor of Creative Industries. Doctor of Philosophy candidate, The University of Queensland. Program Manager – Integrated Care, Children’s Health Queensland, PO Box 3474 South Brisbane 4101, Queensland, AU; 2 Bachelor of Human Services. Principal Community Services Officer, Department of Child Safety, Youth and Women, PO Box 3022 South Brisbane 4101, Queensland, AU; 3 Bachelor of Nursing, Master of Health Management, Post Graduate Diploma in Clinical Nursing (Child and Adolescent Health). Nurse Manager – Navigate Your Health, Children’s Health Queensland, PO Box 3474 South Brisbane 4101, Queensland, AU; 4 Bachelor of Commerce, Master of Public Health, Doctor of Philosophy. Executive Director Community, Mental Health and Statewide Services, Children’s Health Queensland, PO Box 3474 South Brisbane 4101, Queensland, AU; 5 Registered Psychiatric Nurse, Master of Health Science (Hons), Post Graduate Diploma in Health Service Management, Graduate Australian Institute of Company Directors (GAICD). Health Service Chief Executive, Children’s Health Queensland, and Adjunct Associate Professor, The University of Queensland. PO Box 3474 South Brisbane 4101, Queensland, AU

**Keywords:** child protection, child safety, navigation, integration, paediatrics, Queensland

## Abstract

**Introduction:**

Three peak organisations in Queensland, Australia partnered with consumers and other health and social sector partners to co-design and pilot the first known integrated, health navigation model to improve outcomes for children and young people in care in Australia.

**Description:**

An Organisational Learning theoretical lens has been used to present a narrative case study of findings structured as key learnings from the Navigate Your Health pilot to inform quality improvement, scalability and program sustainability. A developmental evaluation was completed whereby semi-structured interviews, focus groups, surveys, chart reviews, database excerpts and economic modelling was completed alongside project documentation analyses to create an evaluation framework.

**Discussion:**

Findings highlighted the agency partners’ drive to foster a more integrated and person-centred approach to care. The pilot’s aim of improving health outcomes for a vulnerable population were achieved through a co-designed process which provided additional insights regarding partnerships, improvement, scalability and sustainability.

**Conclusion:**

Inter-agency responses to system fragmentation provide significant organisational learning opportunities. System integration is achievable through strengthened partnerships that can be sustained beyond a pilot phase to improve health outcomes for vulnerable/priority populations.

## Introduction

The evidence linking child abuse, neglect and trauma to significant and enduring health needs is unequivocal [1]. In the Australian context, three landmark inquiries into the Queensland Child Protection system [2,3,4] revealed that children who are removed from their birth families and placed in care have poorer health outcomes than their peers. At a national level [5,6,7] children in care have less routine health checks, leading to an under-diagnosis of conditions and a lack of access to services.

The most recent inquiry in 2013 [4] recommended that ‘in accordance with the elements of the National Clinical Assessment Framework for Children and Young People in Out-of-Home Care (NCAF) [8], the Department [of Child Safety, Youth and Women], in conjunction with Queensland Health, should ensure that every child in out-of-home care receives a Comprehensive Health and Developmental Assessment completed within three months of placement’ [4].

During late 2016, executive and operational leads from Children’s Health Queensland Hospital and Health Service (CHQHHS) [9] and the Department of Child Safety, Youth and Women (DCSYW) [10] acknowledged the need for systemic reform and committed to addressing this fragmentation at a systems level. After a comprehensive consultation and co-design process to develop the model which lasted approximately eighteen months, joint funding was secured, and the partners launched the Navigate Your Health Program (NYH) as a two-year pilot within the Brisbane District from January 2018 until December 2019.

The NYH model used health navigator roles to coordinate health and developmental assessments for children and young people entering and/or already in care, and connected them with relevant health and support services. The health navigator roles were employed by CHQHHS, and provided an in-reach function by visiting in-scope Child Safety Service Centres to coordinate and manage referrals of children and young people to access general practices, hospitals, Aboriginal medical services and other community health centres as required. The NYH model was designed to facilitate eligible children and young people entering care to undertake two health checks – a preliminary and a comprehensive health assessment – to ensure that physical, dental, developmental, emotional and mental health screening and assessment occurred. These assessments were completed by a range of healthcare providers, including General Practitioners, child and family health nurses and Aboriginal Medical Services. Informed by the findings of these assessments, the Health Navigator would then develop a Health Management Plan to support coordination of the identified healthcare needs of each child or young person for the following twelve months in partnership with the child’s support network.

The objective of the pilot was to test the model of care to improve health and wellbeing outcomes of children in care within the Brisbane District. It had been co-designed with children and young people, families, carers, advocates and staff from both health and child safety sectors and non-government service providers [11]. CHQHHS and DCSYW then partnered with the Aboriginal and Torres Strait Islander Community Health Service – Brisbane (ATSICHS Brisbane) [12], consumer representatives and other primary healthcare and non-government organisational partners to mobilise the pilot.

NYH’s inter-agency, co-design and consumer engagement strategy was driven by CHQHHS’s approach to integrated care [13] and the Queensland Government’s drive to align shared values. The approach centred on working in partnership with children and young people, families, carers and advocates, to maximise benefits to consumers and respective agencies through the integration of the health and welfare systems [14,15].

Both CHQHHS and DCSYW, as the lead agencies, pooled funding for CHQHHS to appoint four multidisciplinary health navigator positions. The positions were to support children through the NYH model and pathway, inclusive of health assessments, prioritised referrals and healthcare coordination; with key timepoints in an annual review cycle designed to measure the improved health outcomes of this new intervention [16].

NYH was the first known model in Australia specifically designed to implement an inter-agency systems response that supported improving health outcomes for children in care. CHQHHS and DCSYW hypothesised that NYH’s innovative approach to bolstering system integration would also serve as an exemplar for growing inter-agency partnerships that could address the pervasive system fragmentation in Queensland’s child protection sector [2,3,4,15,17].

The NYH pilot responded to a gap in knowledge regarding how inter-agency responses can effect improved health outcomes within a vulnerable population [4,18,19]. This paper sets out a case study detailing the organisational learning process, and the impact of system integration in improving health outcomes during the pilot study [18,20]. An Organisational Learning framework approach incorporating a phenomenological lens has been employed to illustrate the lessons learned. Readers will gain insights and knowledge that can support implementation of interpreted models in other contexts that can be applied to support responses that improve health outcomes for vulnerable/priority populations.

The pilot illustrated that better inter-agency response to system fragmentation provides significant opportunities for organisational and system transformation. NYH served as a proof of concept for system integration that is achievable through strong partnerships that when nurtured and sustained beyond the pilot phase can result in improved health outcomes for children and young people in care.

## Ethical approval, if appropriate

The health services evaluation for the Navigate Your Health pilot was approved by the Children’s Health Queensland Human Research Ethics Committee under reference number: HREC/18/QRCH/185.

## Description of the care practice

### Using Organisational Learning Theory to showcase this integrated care innovation

Organisational learning is a process whereby organisational improvement activities occur over time, providing employees within the organisation(s) with opportunities to gain new experiences and create knowledge [21,22]. This new knowledge is then transferred within and beyond the organisation to achieve enhanced outcomes [20,21,22,23].

An organisational learning theoretical framework has been used to illustrate the NYH pilot’s co-design, partnerships and implementation as a series of concurrent events and activities which led to improvements in health outcomes for children in care. These learnings highlight key milestones and junctions that can be replicated, measured and used as quality improvement indicators in other integrated care contexts [18,20,24,25]. The focus of the NYH organisational learnings described in this case study are centred around CHQHHS and DCSYW as the organisational initiators of the integrated care initiative in partnership with ATSICHS Brisbane.

CHQHHS is a state-funded tertiary and quaternary health provider, governed by a Board of Directors, and is funded primarily through service agreements with the Queensland Department of Health. It is a specialist statewide hospital and health service dedicated to caring for children from across Queensland and northern New South Wales [26].

DCSYW is the Queensland Government’s lead agency for child protection and adoption services [10,27]. It is dedicated to protecting children and young people who have been harmed, or are at risk of harm and supports the delivery of services to build families’ capacity to care for and nurture their children [27]. DCSYW workforce operate through the administration of the *Child Protection Act 1999 (Qld)* and the *Adoption Act 2009 (Qld)* and work closely with non-government and government partners in the delivery of child protection services across Queensland [27].

**Figure 1:**
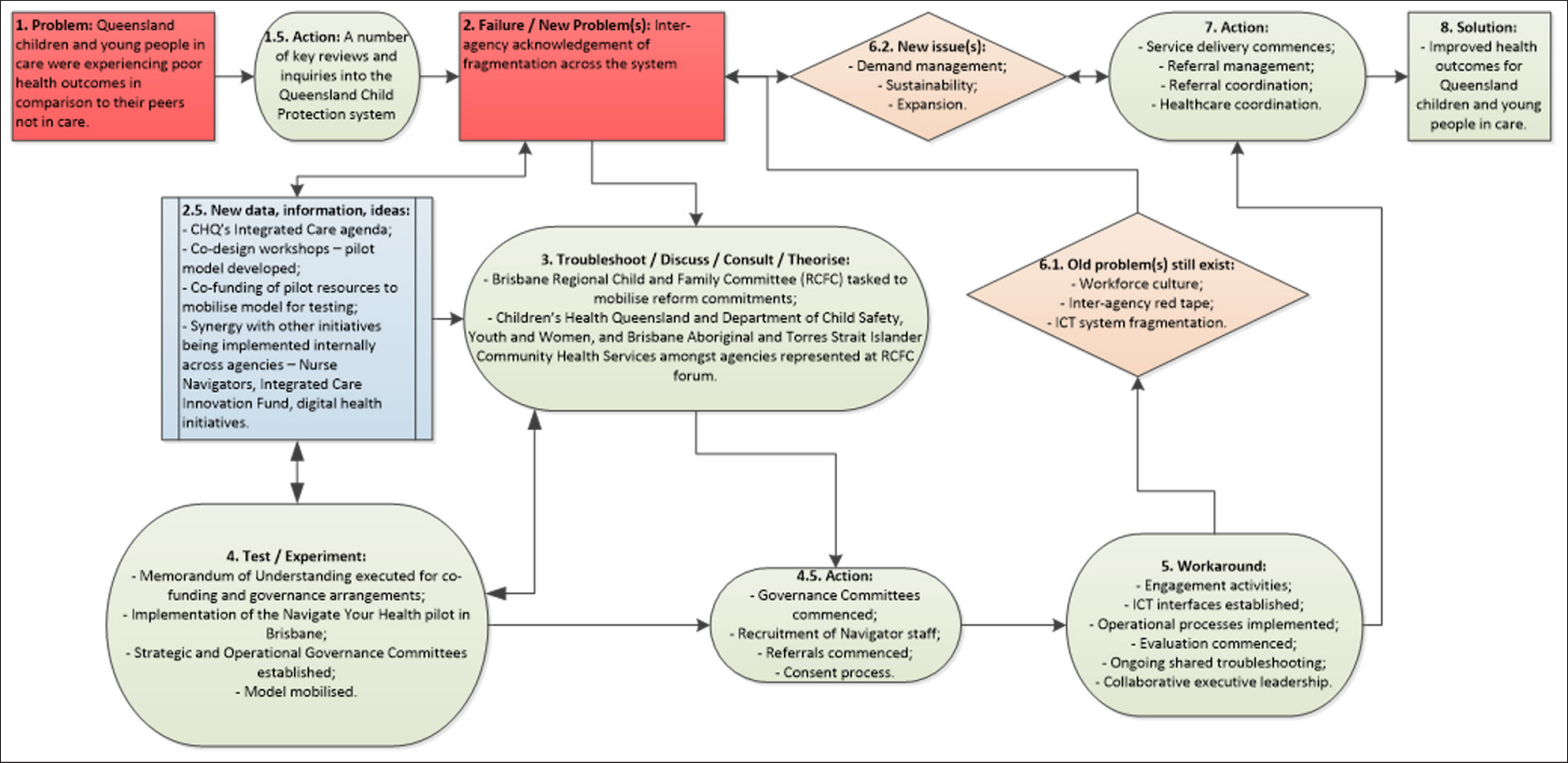
Navigate Your Health – organisational learning theory in use (adapted from Argyris and Schon, 1996) [22].

Collectively, these two organisations were well-positioned to effect strategic and operational change through the NYH pilot by championing the needs of children and young people in care. This included mobilising and implementing changes to work practices within each agency to improve health care outcomes for children and young people in care. ***Figure 1*** below illustrates the NYH pilot’s organisational learning theoretical process as non-linear milestone junctures that provided learning opportunities for the organisations throughout the pilot [22].

Contrary to more espoused theory, that learning and problem solving is a simple, linear process (from problem identification, to action, to solution), the NYH pilot’s organisational learning was not a linear, end-to-end process [22]. In fact, the pilot experienced several experiments and workarounds with some old problems continuing to persist. The following sections of this paper detail the milestones that are illustrated in ***Figure 1***.

The pilot was evaluated over two years using a developmental approach concurrent with implementation [28]. A multiple method design, including quantitative and qualitative data was used to evaluate the design, implementation and short- and medium-term outcomes of NYH. Quantitative data was obtained through audits of CHQ and DCSYW electronic record systems and the NYH SharePoint database (up until August 2019). Qualitative data was collected through a combination of methods including surveys, focus groups, semi-structured interviews, journey maps, literature reviews and desktop audits.

### The Problem, Actions and Failures

Child protection is defined as circumstances where it has been evidenced that the state government must intervene to protect children [29,30]. In Queensland, in circumstances when it is unsafe for the child to remain at home, they are taken into the custody of the DCSYW in line with state legislation to ensure their safety [29,30].

Research consistently shows that children and young people in care are likely to have poorer health and wellbeing outcomes than those who are not in care, including poorer physical, developmental, behavioural and mental/emotional health [1,5,6,31,32,33]. These issues have been directly associated with high levels of system fragmentation between health and welfare agencies, and workforce factors such as high rates of attrition amongst child safety staff [31,33]. The Queensland Child Protection Commission of Inquiry highlighted that children and young people in the care system also have higher rates of earlier onset of sexual activity, higher rates of sexually transmitted infections and higher rates of earlier pregnancy and parenting [4].

During the NYH pilot period, there were approximately 9,107 children in care across Queensland, including 3,832 Aboriginal and/or Torres Strait Islander children [16]. Of this figure, approximately 706 children and young people in care were located within the Brisbane District who were eligible for referral during the pilot period (2018–19) [16].

### Problem-solving, Information Gathering, Pilot Implementation

In October 2016, the Brisbane Region Child and Family Committee (RCFC), an inter-agency governance forum chaired by DCSYW, workshopped the theme of ‘improving health outcomes for children and young people in out of home care’. The RCFC’s focus was drawn from the Queensland-wide Reform Leaders Group, a peak state government forum tasked with overseeing the 2013 Commission of Inquiry recommendations [4], which specifically included this theme. At this meeting, the Brisbane RCFC determined that its efforts throughout 2017/18 under this priority area would include a focus on the development of a best practice model for the health screening of children entering, and currently in care in the Brisbane District. The evidence base for the model’s design as best practice for Queensland was informed by the National Clinical Assessment Framework for Children and Young People in Out-of-Home Care [8], CHQHHS’s Integrated Care agenda [13], and cumulative expertise of the co-design workshop participants.

All partners acknowledged that Aboriginal and Torres Strait Islander children and young people, particularly those in care, experience higher rates of poor health outcomes [6] than their non-Indigenous peers. With this evidence in mind, the RCFC determined the need for an Aboriginal and Torres Strait Islander specific lead agency and approached ATSICHS Brisbane to partner and co-lead the development of the NYH model.

The pilot began with an eighteen-month co-design process. This was led by CHQHHS, DCSYW and ATSICHS Brisbane with staff and partners – including non-government agencies, Primary Health Networks (PHNs) [34], children and young people and foster carers. Stakeholders contributed experience to assist in the development of a best practice model. The NYH pilot model is detailed below in ***Table 1***.

**Table 1:** NYH Model overview.


HEALTH SCREENING	REFERRAL COORDINATION	HEALTHCARE COORDINATION

Children and young people would receive a preliminary health check, followed by a comprehensive health and developmental assessment covering the domains of physical, developmental and mental/emotional health.	The most appropriate pathways for the child/young person would be determined – dependent on the outcome of the preliminary and comprehensive assessments, age, cultural status, disability status, care and health history. Referrals to required services or additional assessments would be progressed and be monitored to ensure the timeliness of follow up and the development of a Health Management Plan.	Children and young peoples’ healthcare would continue to progress in an integrated way, and in line with the recommendations of their Health Management Plan. A higher emphasis and priority for meeting their healthcare needs would be in place. The child’s Child Safety Officer and other roles in the child’s support network would be provided with education to build their health literacy in supporting healthcare coordination.


For the DCSYW, the pilot scope encompassed the Brisbane District geographic boundaries only. Children and young people who were case managed by seven (7) Child Safety Service Centres (Alderley, Fortitude Valley, Chermside, Mt Gravatt, Stones Corner, Inala and Forest Lake) and residing within the Brisbane District, were included in the pilot. For CHQHHS, the pilot’s referral catchment was aligned with that of DCSYW’s Brisbane District.

The NYH model was designed to improve the health and wellbeing outcomes of children in care in the Brisbane District. The model defined the process and pathways for initial health screening, subsequent comprehensive health and developmental assessments, the provision of ongoing healthcare coordination needs, and was supported by the creation of four dedicated Health Navigator positions to be occupied by nursing and allied health professionals.

The co-design process culminated in the endorsement of a proposal to the state government to progress as a jointly funded pilot to test the model of care. Each government partner allocated an in-kind project support team. CHQHHS employed the navigation staff to operationalise the model and integrate the pilot with other complementary innovations. The complementary innovations included CHQHHS implementing priority access to paediatric services for children and young people in care, and a digital innovation project which developed a suite of healthcare assessment and health pathway decision-making algorithms for healthcare providers to use specific to children and young people in care [19].

The pilot was governed under a formalised service agreement between both financial parties (CHQHHS and DCSYW). Strategic and operational governance committees provided oversight to ensure multi-agency stakeholders, decision-makers and consumers maintained engagement and oversight for the duration of the pilot implementation and evaluation phases.

Along with the previously mentioned inquiries [2,3,4], the timing of the pilot also responded to and aligned with several strategic priorities of the Queensland’s public service agencies highlighted in the *Our Future State: Advancing Queensland’s Priorities* report; including prioritising children’s immunisation rates, wellbeing prior to commencing school, keeping Queenslanders healthy, being a responsible government and closing the gap in health outcomes for Indigenous Queenslanders [14,35].

The executive sponsors from each agency capitalised on these strategic priorities at the time to champion collaborative and non-traditional approaches throughout the pilot across a variety of domains including recruitment, information sharing, communication and engagement, resourcing and investment. There was a deliberate intent amongst the executive sponsors to use the pilot as a catalyst to break through historical silos that pre-dated the pilot and contributed to the system fragmentation identified during the previous inquiries. These imaginative approaches facilitated many learning opportunities across the organisational partners and contributed to positive outcomes highlighted in the evaluation [16]. Key examples included are listed in ***Table 2*** below.

**Table 2:** Organisational Learnings.


APPROACH EXAMPLES	ORGANISATIONAL LEARNINGS

**Recruitment:** Young people and foster carers consulted in role description development for Health Navigator roles; Young people with lived experience in care involved in recruitment shortlisting and interview panels for Health Navigator roles; Representatives from each partner agency involved in recruitment shortlisting and interviewing for Health Navigator roles.	Central focus on what characteristics young people value in health workforce; Role modelling of co-design process to interview candidates; Mindfulness of the importance of incorporating the consumer’s voice in decision-making processes.

**Consent and Information Sharing:** Bespoke program consent forms developed; Routine referralprocesses and documentation processes agreed by both agencies; Ongoing quality improvements madethroughout the pilot where required to facilitate and sustain changemanagement activity.	Iterative process to consult with both agencies’ legalservices teams, health and child safety staff to develop consentform; Mindfulness of incorporating relevant legislation from both Child Safetyand Health sectors; Consistent referral documentation collation and submission processesto initiate and complete a successful referral into the pilot.

**Communication and Engagement:** Information flyers co-branded with all agencies’ logos to showcase partnership, written in low health literacy language; Joint media and engagement opportunities to promote the pilot, joint presentations delivered in partnership by agency partners.	Ability to raiseprofile of pilot across multiple stakeholder forums to socialise thebenefits to children and young people; Mindfulness of communicating thebenefits of the pilot to stakeholders with low literacy levelsto ensure their understanding and avoiding jargon; Public displays ofunity and commitment to improve.

**Dedicated Project Management Resourcing:** Strategicand Operational Governance Committees; Frontline culture and workforce change agentin Child Safety and Health systems; Active seeking of eligiblereferrals from across in-scope Child Safety Service Centres to maintaintraction in referring children and young people to the pilot; and a developmental evaluation of the pilot.	Shared governance meant key decisions were consulted upon and discussed in open forums with relevant stakeholders providing subject matter expertise, and executive sponsors’ endorsement; Supported change amongst health providers to prioritise this vulnerable population for care above general triage ratings; Dedicated project resources were essential to sustain practice change and referral momentum in Child Safety; Developmental evaluation was essential to document and analyse the pilot’s outcomes and identify recommendations for ongoing improvements throughout the pilot.

**Co-Investment:** Project Management roles – in-kind; Health Navigators – jointly-funded.	Project resourcing was essential to facilitate and navigate organisational complexitiesbetween partner agencies; Shared financial investment sustained each partner agency’sexecutive sponsorship and championing throughout the pilot; Set a precedentand role modelled successful approach for future opportunities to jointlyinvest in improvement initiatives.


The pilot Health Navigator staffing commenced in January 2018, with referrals commencing in March 2018, through a staged prioritisation of children and young people as illustrated in ***Table 3*** below. They were co-located across the seven in-scope Child Safety Service Centres throughout the Brisbane District and CHQHHS hospital and community health facilities. Health Navigators were responsible for supporting children though the NYH model and relevant healthcare pathways, inclusive of systems navigation, data collection, documentation and coordination processes; with key timepoints in an annual review cycle as summarised in ***Table 1*** above.

**Table 3:** Prioritisation Criteria for referral to the NYH pilot.


NEW TO CARE	ALREADY IN CARE	SUBSEQUENT PRIORITY CRITERIA FOR REFERRAL

Child or young person entered care for the first time, during the pilot period 2018–2019 (prospective control group)	Child or young person has already been residing in care prior to pilot commencing (retrospective comparison group)	Identify as being of Aboriginal and/or Torres Strait Islander origin; Are under six years of age – to support earlier intervention; Are aged 15 years and over – to support earlier transition for adolescent and young adult services; Entered out-of-home care due to neglect concerns; Are in contact with the youth justice system; Are from culturally and linguistically diverse backgrounds; Have not seen a General Practitioner in the previous 6–12 months; Have unaddressed or poorly understood health or development concerns as recognised by Child Safety staff; Are not up to date with immunisations; and/or Are siblings of the above cohort.


It was anticipated that within the first year of operation, each of the four Health Navigators would commence the healthcare assessment, referral coordination and healthcare coordination process for 100–150 of the approximate 706 children within the Brisbane District, taking into account the variability in the complexity of their health needs.

Over the course of the two-year pilot, 569 children and young people in care from across the seven in-scope Child Safety Service Centres were referred to the program. ***Table 4*** below provides a demographic profile of the children and young people that were referred during the pilot.

**Table 4:** Demographic Profile of Children and Young People referred during the pilot.


	NUMBER	PERCENTAGE

**Age**		

0–5 years	254	44.64%

6–12 years	214	37.61%

13+ years	101	17.75%

**Gender**		

Female	282	49.56%

Male	287	50.44%

**Indigenous Status**		

Aboriginal and/or Torres Strait Islander	152	26.71%

Non-Indigenous	401	70.47%

Unknown	16	2.82%

**Care status**		

Already in care	356	62.57%

New to care	213	37.43%


While significant time and resources were invested in ensuring the lead up to and implementation phase of the pilot went smoothly some obstacles, both old and new, required the organisational partners to take actions and resolve collaboratively. In parallel to the pilot, the developmental evaluation was designed and completed to investigate whether the NYH model would achieve improved health outcomes for children and young people in care [16,28,36].

### Old and New Problems

The Strategic and Operational Governance Committees identified and oversaw the management of a number of existing and emergent problems encountered during the NYH pilot implementation. Three key problems were anticipated and remained pervasive during the pilot:

1. **Workforce culture and resistance** to change was an ongoing barrier to implementation. The partners had to shepherd static employees who were anxious, unfamiliar and/or insubordinate with the proposed partnership and changes in practice [20]. Motivations for resistance were associated with a perceived increase in workloads, loss of control in decision-making and increased transparency across previously siloed teams. The executive leads identified and persevered with implementing both system change and practice change.
2. **Protracted bureaucratic processes** associated with financial and industrial agreements often impacted the timeliness in which the NYH pilot’s implementation activities occurred [15,17]. This was due to the time taken in administrative processing of agreements and correspondence being executed by relevant delegates in each organisation, and industrial consultation and negotiations regarding the utilisation of clinical workforce roles practicing in new contexts during the pilot. Clinical and operational supervision and delegated authority practices also varied between partner agencies, requiring time to negotiate terms for documentation in operational guidelines and processes. Specifically, for CHQHHS, clinical governance and quality assurance processes also required an investment of time as part of the organisation’s accreditation responsibilities as a provider of health services.
3. **Information, communication and technological system fragmentation** was a pre-existing challenge, with each partner agency’s information systems being designed as bespoke for their business as usual operations and secured from third party access.

These three key challenges have been consistently identified as common impediments to innovation across public service agencies [15,37]. While these problems remained present during the pilot, workarounds were identified, negotiated and reviewed to ensure the pilot’s potential would be realised while maintaining compliance with legislative standards [16]. These workarounds presented opportunities for both organisations to learn new and innovative ways to collaborate and integrate more effectively to achieve the mutual goal of improving health outcomes for children and young people in care.

New issues also emerged during the pilot which required collective consideration and oversight by the governance committees to ensure the pilot could proceed as expected. The three key issues that emerged:

1. **Demand management** – the Child Safety project lead actively scouted for eligible referrals and drove operational systems change at the Child Safety Service Centre level throughout the pilot. This was essential to encourage and facilitate practice change amongst the Child Safety workforce who initiated each referral for NYH as a new service offering. The Child Safety project lead role also routinely provided in-service education sessions, communicated positive outcomes and fed-back on opportunities and challenges to ensure the uptake of Health Navigator resourcing was utilised equitably across in-scope Child Safety Service Centres to benefit children and young people across all sites.
2. **Sustainability planning** was a key focus for the partner organisations. Given the economic context in which public services operate in Queensland and the novel nature of the pilot, there was ongoing focus to ensure the roles remained economically viable and were achieving outcomes. Demand for NYH’s referral eligibility to evolve and change during the pilot came from a variety of internal and external stakeholders. To manage this, the partner organisations retained joint decision-making on the eligibility criteria at the governance committees’ level to ensure that fidelity of the model was protected during the pilot to test the intervention’s effectiveness. It was considered that the Health Navigator roles as a new workforce needed sufficient and protected time during the pilot to deliver the health intervention illustrated in ***Table 1*** above to demonstrate this new intervention could improve outcomes when compared to the status quo [16].
3. **Expansion planning considerations** emerged prior to the pilot term concluding, and in mid-2019, a workshop was held to explore the feasibility of scaling the model to additional locations in Queensland and also applying the NYH model to support the Youth Justice population. This was as a result of early successes, positive feedback from stakeholders and partners, and improved outcomes being achieved. As a result, inter-agency negotiations regarding future resourcing requirements were brought forward earlier than anticipated and were agreed upon before the final evaluation outcomes of the pilot were known.

These problems, both anticipated and emergent, required an investment of time and provided learning opportunities at strategic and operational levels. Both partner organisations remained committed to the ongoing success and were sufficiently satisfied with the outcomes achieved to guarantee ongoing investment in scaling the pilot to additional locations and also to the Youth Justice population from early 2020. As a result, the partners were well-positioned to take these cumulative lessons learned forward and inform the ongoing delivery of services beyond the pilot phase.

### Action and Solution

The evaluation data collection period lasted from when referrals commenced in March 2018 up until August 2019. During this time, 569 children and young people had been referred to the NYH pilot from across the seven in-scope Child Safety Service Centres. The evaluation findings [16] were delivered in February 2020, and evidenced that the NYH model demonstrated:

- Delivery of enhanced child, young person and family-centred care;
- Delivery of safe, high quality, integrated, and evidence-driven health care coordination;
- Improved engagement with children, young people, families and/or carers in health decision-making;
- Improved health literacy and system awareness for children, young people, families and/or carers, and the program partner workforce;
- Improved health outcomes, particularly in the domains of immunisation status and routine oral health checks;
- Increased knowledge and understanding of other complimentary service components across the system that can interface with NYH;
- Promotion of increased consistency and streamlining of service delivery across child safety and health services via NYH;
- Enhanced partnership, practice innovation, service development, evaluation and review processes across partnering teams to improve health care outcomes for children and young people in care or engaged with Child Safety; and
- The intervention was provided at a mean cost of $35 per child, per week of enrolment (inclusive of all the labour, capital and consumables). This represented a modest investment in healthcare coordination on a per child basis. It was also noted that these costs could likely be revised down following initial once-off costs borne from the pilot implementation.

As a result of the evaluation, the partner organisations were satisfied that the health outcomes for children and young people in care had been improved as a result of the NYH pilot.

## Discussion

The main finding of this study highlights that organisational learning, as evidenced by the NYH pilot implementation, was not a linear process. In this Queensland example, inter-agency partners embarked on piloting an Australian-first, co-designed, evidence-based model to improve health outcomes for children and young people in care. The pilot achieved positive results and went on to be scaled prior to formal evaluation findings being released, based on cumulative evidence and feedback of success stories from stakeholders.

These learning outcomes experienced during the NYH pilot were a result of collective troubleshooting and recognised that neither the inter-agency partners or the consumer group were homogeneous populations, with the model supporting an array of learning interventions across the health continuum to address the diverse needs of vulnerable children and young people.

The unique cross-agency collaboration combined with consumer co-design and implementation had a considerably positive impact on the delivery of the NYH pilot program. The implementation of NYH demonstrated organisational commitment to change within both the health and child safety systems to provide healthcare coordination in a way that worked for service users and enhanced system responsiveness. The collaboration was established through consistent and documented processes and pathways, which indicated the likelihood of ongoing successful outcomes in the future.

The NYH model delivered on the core elements of the National Clinical Assessment Framework including for a care coordinator health officer role to ensure the completion of health assessments upon entry to care, the development of a health management plan and follow-up monitoring in accordance with clinical needs [8]. The NYH pilot learnings contributed greatly to an enhanced understanding of the health needs of children in care in the Brisbane District, and made progress towards addressing the substantial, complex and longstanding unmet health needs of this vulnerable population.

As a result of the NYH pilot, there was a high number of children in care who received a Comprehensive Health and Developmental Assessment and had a health management plan in place to guide the actions required to address their identified health needs.

The developmental evaluation’s surveys, focus groups and interviews found that overall satisfaction with NYH of consumers was high, with children and their birth families/carers experiencing increased communication and information sharing. The consistency of care experienced, specifically the Health Navigator role and service provider(s) completing the health assessment, was likely to have long-term benefits with regard to continuity of care via the establishment of positive relationships with healthcare professionals and improved health outcomes.

Through NYH, short- and medium-term health outcomes in relation to improved immunisation coverage were achieved, and higher levels of engagement in preventative health measures, such as routine oral healthcare checks. Carers, in particular reported significant improvements in health outcomes for children referred to NYH.

The environmental context across NYH’s inter-agency partnerships provided organisational learning through identified enablers and barriers to the pilot’s implementation success in Queensland to integrate and improve healthcare outcomes for children and young people in care.

One limitation of this case study was that there was no comparison model to benchmark against at the time of implementation. However, future studies in other contexts may provide new knowledge where the NYH model can be applied and tested in different communities and/or augmented to suit new priority populations. This case study presents new knowledge in the realm of organisational learning processes when implementing innovative pilots to achieve more integrated care for children and young people. It showcases a positive example from the Queensland context, and highlights points of focus that other project teams and researchers can consider when conceptualising similar pilot initiatives.

## Lessons learned

- System integration achieves improved health outcomes;
- Consumer engagement is essential to implement sustainable healthcare interventions;
- Innovation presents a compelling narrative for ongoing investment in change;
- Co-designing innovation requires sustained commitment long-term; and
- Shared governance and investment are essential to effect and sustain inter-agency change.

## Conclusion

To conclude, this case study illustrated the non-linear organisational learning processes and evolving environmental context across NYH pilot’s implementation. Through the inter-agency partnership, there were key enablers and barriers to the NYH pilot’s co-design and implementation successes in Queensland which integrated and improved healthcare outcomes for children and young people in care. The pilot model’s co-design and governance contributed significant and ongoing organisational learning opportunities to refine and evolve the model, which led to ongoing investment and scaling prior to the pilot concluding.
